# Sizing single nanoscale objects from polarization forces

**DOI:** 10.1038/s41598-019-50745-5

**Published:** 2019-10-02

**Authors:** H. Lozano, R. Millán-Solsona, R. Fabregas, G. Gomila

**Affiliations:** 1 Nanoscale Bioelectrical Characterization, Institute for Bioengineering of Catalonia (IBEC), The Barcelona Institute of Science and Technology (BIST), Baldiri i Reixac 11-15, 08028 Barcelona, Spain; 2 Departament d’Enginyeria Electrònica i Biomèdica, Universitat de Barcelona, Martí i Franqués 1, 08028 Barcelona, Spain

**Keywords:** Characterization and analytical techniques, Imaging techniques

## Abstract

Sizing natural or engineered single nanoscale objects is fundamental in many areas of science and technology. To achieve it several advanced microscopic techniques have been developed, mostly based on electron and scanning probe microscopies. Still for soft and poorly adhered samples the existing techniques face important challenges. Here, we propose an alternative method to size single nanoscale objects based on the measurement of its electric polarization. The method is based on Electrostatic Force Microscopy measurements combined with a specifically designed multiparameter quantification algorithm, which gives the physical dimensions (height and width) of the nanoscale object. The proposed method is validated with ~50 nm diameter silver nanowires, and successfully applied to ~10 nm diameter bacterial polar flagella, an example of soft and poorly adhered nanoscale object. We show that an accuracy comparable to AFM topographic imaging can be achieved. The main advantage of the proposed method is that, being based on the measurement of long-range polarization forces, it can be applied without contacting the sample, what is key when considering poorly adhered and soft nanoscale objects. Potential applications of the proposed method to a wide range of nanoscale objects relevant in Material, Life Sciences and Nanomedicine is envisaged.

## Introduction

Determining the physical dimensions (width and height) of manufactured and natural nanoscale objects, such as nanoparticles, nanowires, nanotubes, nanofibers, macromolecular protein complexes, viruses, liposomes, nanodroplets, etc. is of key importance in many areas of science and technology, such as nanocatalysis^1^, nanofiltering^2^, and nanomedicine and nanotoxicology^3–6^. There exist several characterization techniques to determine the dimensions of nano-objects^7,8^. Examples include ensemble techniques, such as Dynamic Light Scattering^9^ or Nanoparticle Tracking Analysis^10^, and single nanoscale object techniques such as Transmission Electron Microscopy^11^ and Atomic Force Microscopy^12^. Among single nano-object sizing techniques, Atomic Force Microscopy (AFM)^13^ is one of the more used and versatile ones since it can address the three spatial dimensions of the nano-objects, it can be applied to nano-objects of any material type (metallic and insulating, organic and inorganic), and it can be used in any environmental condition (air, liquid, vacuum)^14–17^. AFM can determine the height of the nano-objects on a substrate with nanometric precision in a relatively straightforward way. Concerning the width, AFM can provide a good estimation of it, although some post-processing of the data is necessary to account for tip dilation effect and other factors^18^. Tip dilation effects are due to the finite size of the measuring tip, and they tend to broaden the measured topography. To subtract these effects, geometric deconvolution methods combined with tip geometry calibration methods have been developed^19–25^, whose accuracy varies largely depending on the nature, geometry and size of the nano-objects and of the measuring probes. In general, the width is determined by AFM with a smaller accuracy and reproducibility than the height.

Despite its wide use, sizing single nanoscale object by AFM faces difficulties for the case of poorly adhered or soft nano-objects. For poorly adhered nano-objects the AFM tip can induce lateral displacements of the object during the image acquisition, which can further broaden the measured topography^26,27^. Moreover, for soft samples, the lateral and vertical forces exerted by the tip on the nano-object during the image acquisition can deform the sample, and, then, can impact on the measured topography^28,29^. Therefore, sizing poorly adhered or soft samples still constitutes a challenge for AFM.

Here, we present an alternative scanning probe microscopy method to size single nanoscale objects that overcomes the above-mentioned limitations for poorly adhered or soft nano-objects. The method is based on the measurement of the nano-objects electrical polarization, rather than on the measurement of its topography, and on the extraction of the physical dimensions from the electric polarization by using a specifically designed multiparameter quantification algorithm. The electric polarization of the nano-objects is measured by Electrostatic Force Microscopy (EFM), while the quantification multiparameter extraction algorithm generalizes to multiple parameters the single parameter procedures used to quantify the dielectric constant of nano-objects by Scanning Dielectric Microscopy^30–33^. The proposed EFM method, being based on the measurement of long-range polarization forces, can be applied without contacting the sample at any moment and, hence, is fully compatible with poorly adhered or soft nanoscale objects. Here, we validated the method with silver nanowires, and, as an application, we considered the case of a macromolecular protein complex (bacterial polar flagella), which constitutes an example of a fragile and low polarizable sample.

## Results

### Characterization of the silver nanowire test sample by conventional AFM, TEM and SEM imaging

**Figure 1:**
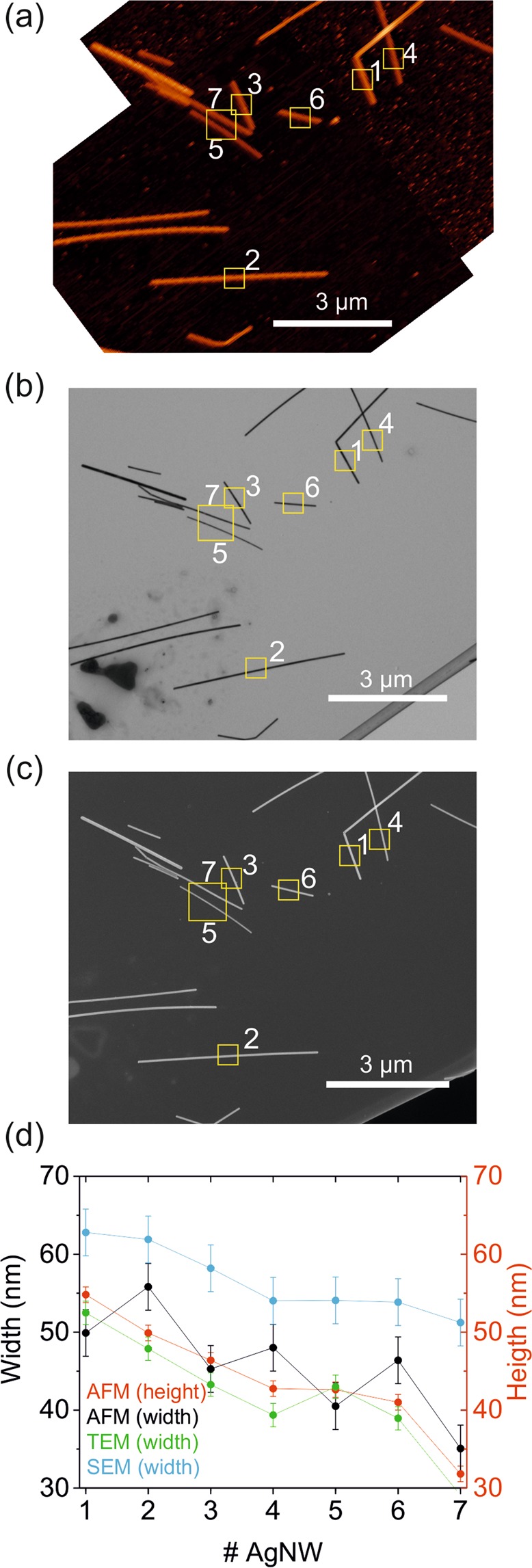
**(a**) AFM, (**b**) TEM and (**c**) SEM images of the same set of silver nanowires deposited on a TEM finder grid. The seven numbered NWs are those analyzed explicitly. (**d**) Height (red symbols, right axis) and (deconvoluted) width (black symbols, left axis) of the numbered NWs obtained from zoom-ins AFM images. Green and blue symbols represent the widths of the same NWs measured from zoom-in TEM and SEM images. AFM tip geometry (obtained from an EFM approach curve): apex radius R = 31 ± 2 nm and half cone angle θ = 25 ± 1°.

Before presenting the new EFM based method, we have analyzed the test sample to be used in its validation (~50 nm diameter silver nanowires, AgNWs) with the conventional single nanoscale object sizing techniques, namely, AFM, TEM and Scanning Electron Microscopy (SEM). Figure 1a–c show, respectively, AFM, TEM and SEM images, of a given set of AgNWs deposited on a TEM finder grid (see Materials and Methods and Fig. S1 in the Supplementary Information). Figure 1d shows the heights and (deconvoluted) widths obtained from the AFM images (red and black symbols, respectively), as well as, the widths measured from TEM (green symbols) and SEM (blue symbols) images (see Materials and Methods and Figs S2–S5 in Supplementary Information). For greater accuracy, in all cases, the physical dimensions were obtained from zoom-in images taken on each individual nanowire (see Fig. S2 in Supplementary Information). The error bars in the measured heights and widths represent the standard deviation of five consecutive profiles in the images.

The (deconvoluted) widths obtained from topographic AFM imaging closely follow the widths determined by TEM, within the experimental error (±5 nm). Instead, the SEM widths tend to be systematically overestimated ~ 10 nm, as has been reported earlier^16^. Finally, the heights obtained from the topographic AFM images are very close to the widths measured by TEM, with just a slightly systematic underestimation (~1–3 nm). This fact is consistent with an almost circular geometry of the AgNWs. These results show that AFM topographic imaging combined with the tip deconvolution process implemented here (see Materials and Methods), offers an accurate description of the dimensions of the AgNWs, and hence, it can be used as a good reference method to validate the proposed EFM method.

**Figure 2:**
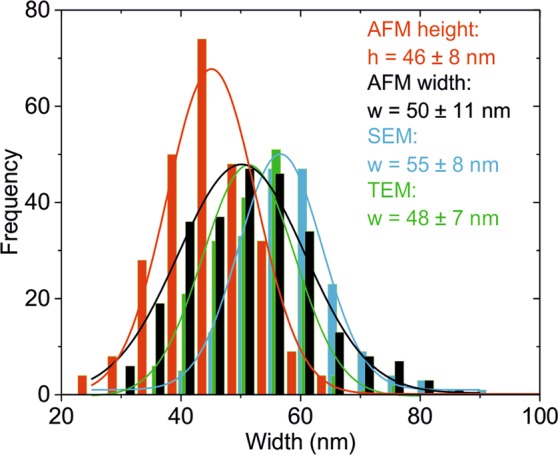
Histogram representation of a population analysis of the dimensions of the AgNWs by different techniques: (red bars) AFM height, (black bars) (deconvoluted) AFM width (N_AFM_ = 134), (green bars) TEM widths (N_TEM_ = 91), (blue bars) SEM widths (N_SEM_ = 91). The lines represent Gaussian fits to the different distributions, giving mean values: h_AFM_ = 46 ± 8 nm, w_AFM_ = 50 ± 11 nm, w_TEM_ = 48 ± 7 nm, and w_SEM_ = 55 ± 8 nm. Tip calibrated geometry for AFM deconvolution analysis: R = 33 ± 1 nm, θ = 11 ± 1°.

This conclusion has been confirmed from a population analysis of the nanowire sample, whose results are shown in Fig. 2 (see Fig. S6 and Table S1 in Supplementary Information). The height and width values show approximately a Gaussian distribution with mean values: *h*_*AFM*_ = 46 ± 8 nm, *w*_*AFM*_ = 50 ± 11 nm (*N*_*AFM*_ = 134), *w*_*TEM*_ = 48 ± 7 nm (*N*_*TEM*_ = 91) and *w*_*SEM*_ = 55 ± 8 nm (*N*_*SEM*_ = 91), corresponding, respectively, to the AFM heights (red bars), AFM (deconvoluted) widths (black bars), TEM widths (green bars) and SEM widths (blue bars). The errors here represent the standard deviation within the population of nanowires obtained from the Gaussian fit to the data. Each nanowire has been measured twice (in different points) to reduce the error of the measurement. Again, the (deconvoluted) AFM and TEM widths give similar results, while the SEM widths tend to be systematically overstimated ~7 nm. Moreover, the AFM height is very close, just slightly smaller, than the AFM or TEM widths, confirming the almost circular nature of the nanowires.

### Sizing single silver nanowires from electric polarization forces

The proposed method consists in measuring the electric polarization of single nano-objects by EFM, and then use a multiparameter quantification algorithm to retrieve the dimensions of the nano-object that best reproduces the measured electric polarization (by assuming the dielectric constant of the object to be known, see discussion). The dependence of the electric polarization of an object on its shape and size is a well-known property of polarizable materials^34,35^. The quantification algorithm to extract the physical dimensions of the nano-object from the measured polarization forces is inspired in the method developed earlier to measure the dielectric constant of nanoscale objects^36^, but generalized to the much more complex situation of multiple parameter extraction (here the width and height). We have analyzed theoretically a similar quantification algorithm in the context of EFM tomographic reconstruction^37^, but it has not been implemented it in practice, yet.

**Figure 3:**
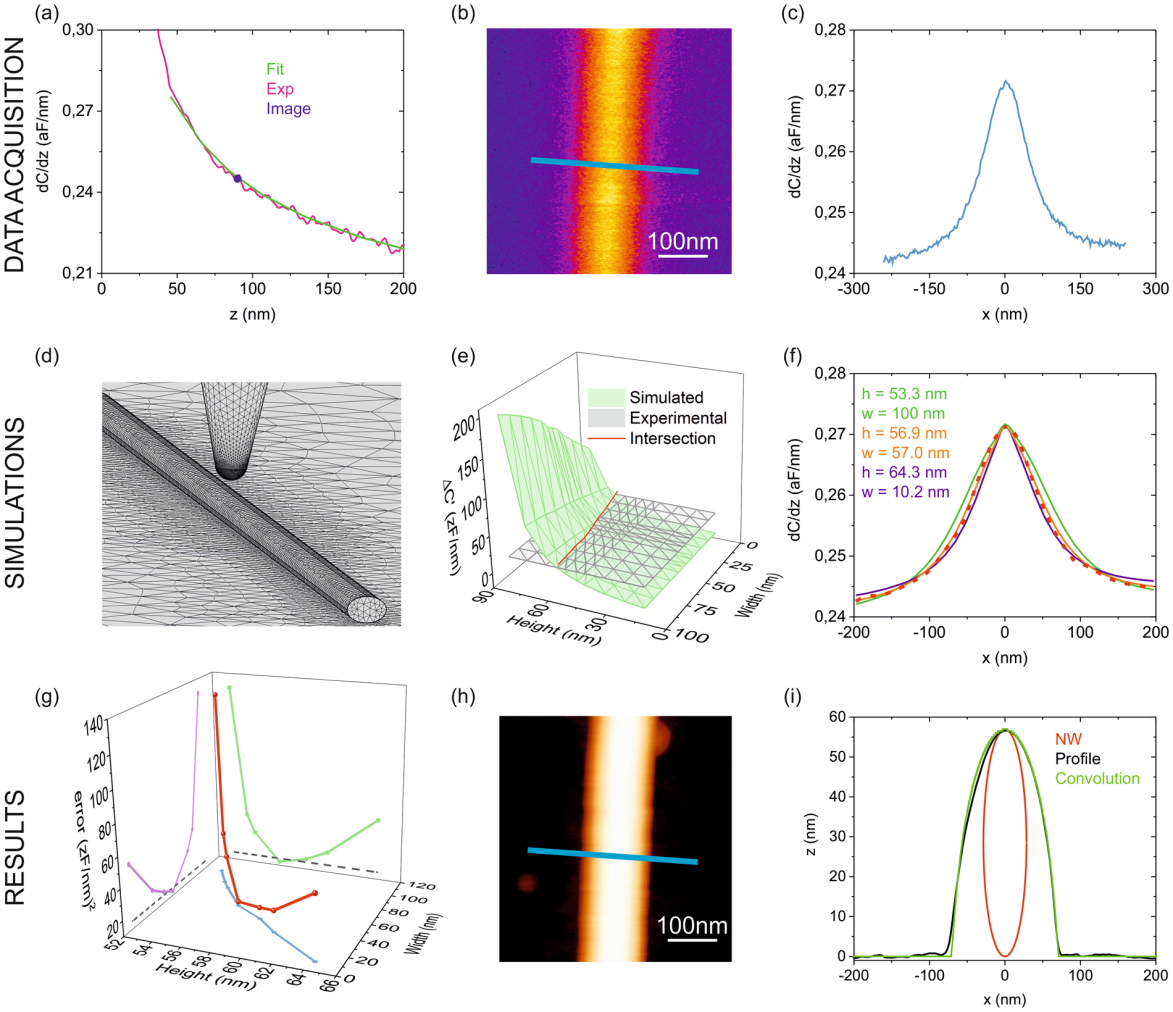
(**a**) Capacitance gradient EFM approach curve on a bare part of the metallic substrate (red line). The green line corresponds to a least square fitting of the theoretical model to determine the tip geometry. It gives R = 42 ± 1 nm, θ = 23 ± 2°, C’_offset_ = 112 ± 2 zF/nm. The blue circle represents the value on the substrate of the EFM image in (**b**) used to determine the tip-sample distance of the EFM image giving z = 87 ± 2 nm. (**b**) Capacitance gradient EFM image measured in constant height mode on a single nanowire. (**c**) Capacitance gradient cross-section profile along the line in (**b**). (**d**) Geometrical tip-nanowire model used for the numerical calculations. (**e**) (Green surface) Calculated maximum capacitance gradient EFM contrast as a function of the width and height of the nanowire. (Gray plane) Experimental capacitance gradient contrast obtained from (**c**), ΔC’ = 28 ± 1 zF/nm. (**f**) (Continuous lines) Example of numerically calculated capacitance gradient profiles corresponding to the couples of height/width values that provide the experimental contrast, compared to the experimental profile (dashed red line). (**g**) (Red symbols) Cumulative error square R^2^ of the calculated profiles as a function of the height and width giving a contrast equal to the experimental one. The purple and green symbols are the projections of the main curve on the lateral planes of the width and height. The position of the minimum gives the dimensions of the AgNW that best fits the experimental profile (h_EFM_ = 58 ± 1 nm and w_EFM_ = 55 ± 9 nm). The dashed lines represent the noise error floor for R^2^. (**h**) AFM image of the same AgNW shown in (**b**). (**i**) Deconvolution of the AFM profile giving w_AFM_ = 57 ± 2 nm *and* h_AFM_ = 59.0 ± 0.5 nm. The error corresponds to five consecutive measured profiles.

The complete procedure is described in Fig. 3 (for details on the EFM measurements and numerical calculations see Materials and Methods). The EFM measurements have been performed on a custom-made AFM/EFM/SEM finder grid, which enables also performing AFM and SEM measurements for validation purposes (see Materials and Methods and Fig. S7 in Supplementary Information). We first calibrate the tip geometry from an EFM capacitance gradient approach curve measured on a bare part of the substrate, as explained elsewhere^30,38^ (Fig. 3a). In the present example, we obtain a tip radius *R* = 42 ± 1 nm, a half cone angle *θ* = 23 ± 2°, and a capacitance gradient offset *C’*_*offset*_ = 112 ± 1 zF/nm. Then, we localize the nano-object of interest from a conventional AFM image (not shown) and acquire a calibrated constant height EFM image (Fig. 3b). From the EFM image we extract a capacitance gradient cross-section profile, which can be eventually averaged with nearby profiles to further reduce the instrumental noise (Fig. 3c). A geometrical model is then built to describe theoretically the electric polarization of the nanoscale object and the electric force acting on the tip (Fig. 3d). In the present case, the model consists of the tip, with the calibrated geometric parameters, and the nanowire, which is assumed to have an ellipsoid cross-section with variable height and width. For simplicity we consider nanowires longer than ~1 μm, for which finite length effects can be neglected^33^. The dielectric constant of the nanowire is assumed to be known (in the present case we assume a metallic nature for the AgNW, which can be modeled by a very large value of the relative dielectric constant, e.g., *ε*_*r*_ = 10^6^). The tip-sample distance in the model is set with the help of the EFM approach curve used to calibrate the tip geometry (Fig. 3a), by using values measured on bare parts of the EFM image (Fig. 3b) (blue circle in Fig. 3a, giving here *z* = 87 ± 2 nm with respect to the substrate). The theoretical model is, then, numerically solved and used to calculate all possible EFM capacitance gradient values at the center of the nanowire (maximum contrast) for different combinations of the height and width of the nanowire (here we swept the width between 10 nm and 100 nm and the height between 10 nm and 60 nm). The calculated maximum capacitance gradient contrast as a function of the width and height of the nanowire is shown in Fig. 3e (green surface). The intersection of this surface of possible EFM contrast values with the experimental measured EFM contrast (here *ΔC’* = 28 ± 1 zF/nm, see Fig. 3c) determines the set of height/width couples compatible with the measured maximum contrast (red line in Fig. 3e). For each couple of possible height/width values, full capacitance gradient profiles are calculated and compared with the experimental one (Fig. 3f). For greater accuracy, in the calculations of the profiles we account for the small tilting of the sample and bending of the cantilever when passing above the nanowire (see Fig. S8 in Supplementary Information). For each calculated EFM profile we determine the cumulative quadratic error *R*^2^ with respect to the experimental EFM profile. By performing this calculation for all possible height/width couples, and plotting *R*^2^ against these variables, we observe the presence of a minimum in *R*^2^ for one of the height/width couples (see Fig. 3g). This couple of values represent the solution sought, since for them the calculated EFM profile shows the minimum error with respect to the experimental one. In the present example we obtained *h*_*EFM*_ = 58 ± 1 nm and *w*_*EFM*_ = 55 ± 9 nm. The error in the width and height of the AgNW has been estimated by setting a noise floor to *R*^2^ determined by the experimental noise (dashed lines in Fig. 3g). We essentially assume that a calculated EFM profiles can be distinguished from the experimental EFM profile if there exists a cumulative quadratic error larger than *R*_*det*_^2^ = *(3/*2 *δC’)*^2^*n, where *δC’* is the experimental noise of the measured profile and *n* the number of points of the calculated profile, and where the value of the minimum of *R*^2^ is roughly *R*_*min*_^2^ = *(δC’)*^2^*n. In the present case, the instrumental noise was *δC’* = 0.5 zF/nm and the number of calculated points per profile *n* = 36, from where *R*_*det*_^*2*^ = 17 (zF/nm)^2^ (dashed lines in Fig. 3g).

We remark that the physical dimensions obtained in the previous analysis have been determined solely from information obtained from the electric polarization measurements, with no input coming from the sample topography. This result shows that it is possible to derive the physical dimension of a nanoscale object from electric polarization measurements alone, provided the dielectric constant of the object is known.

The results obtained from the EFM method can be compared with the ones obtained from conventional AFM imaging on the same nanowire. For the specific AgNW analyzed in Fig. 3, the corresponding topographic AFM image is shown in Fig. 3h. The tip deconvolution analysis of this AFM image (Fig. 3h) gives *w*_*AFM*_ = 57 ± 2 nm and *h*_*AFM*_ = 59.0 ± 0.5 nm. These values are almost identical (within the experimental error) to the ones derived with the EFM method (*h*_*EFM*_ = 58 ± 1 nm and *w*_*EFM*_ = 55 ± 9 nm). We highlight, specially, the excellent agreement between the heights obtained by the two methods, given the relatively small uncertainty. For the widths the agreement is also good, although the uncertainty in the EFM method is somewhat larger than in the AFM method (see Discussion). We have also taken a SEM image of the same AgNW analyzed in Fig. 3 (see Fig. S9 in Supplementary Information), thanks to the use of the custom-made AFM/EFM/SEM finder grid. We obtained for the width *w*_*SEM*_ = 67 ± 2 nm, consistent with the values of the widths obtained by AFM and EFM above, provided one considers the systematic ~7–10 nm broadening effect of SEM images described before.

**Figure 4:**
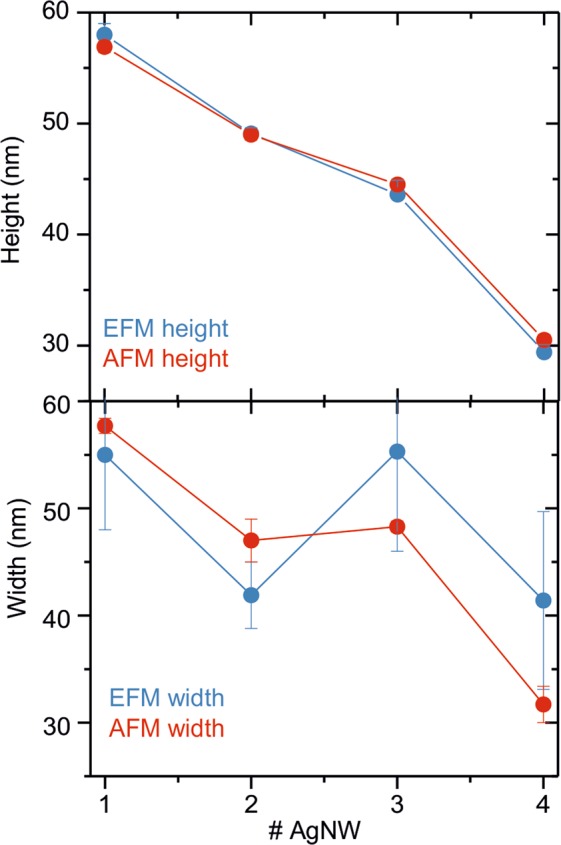
Heights (top panel) and widths (bottom panel) obtained on four different AgNWs with sizes spanning the full range of sizes present in the nanowire sample with the EFM method (red symbols) and with the conventional AFM topographic method (blue symbols).

Similar conclusions have been obtained from different nanowires analyzed, whose sizes span the full range of sizes present in the sample (see Figs 4 and S10–S12 in Supplementary Information). Then, we conclude that, by measuring the electric polarization of nanoscale objects of known dielectric constant, one can obtain its physical dimensions with an accuracy like that obtained from conventional AFM topographic imaging, especially on what concerns the height. The main advantage of the proposed method is that the dimensions of the nano-objects are obtained from images that are acquired in non-contact mode (constant height EFM). Hence, it is expected to provide more reliable values for the case of soft or poorly adhered samples.

### Application to polar bacterial flagella

As an example of application to a fragile sample, we consider the case of bacterial polar flagella, whose dielectric properties have been analyzed by us recently^33^. Bacterial polar flagella are long-thin (~10–20 nm diameter) whip like appendages that bacteria use to move towards the nutrients. They are composed of 11 flagellin protein monomers twisting every ~5 nm^39^. We have determined the dielectric constant of flagella belonging to two different bacterial species, *Pseudomonas aeruginosa* PAO1 and *Shewanella oneidensis* MR-1. We obtained dielectric constant values of *ε*_*So*_ = 4.3 ± 0.6 and *ε*_*Pa*_ = 4.5 ± 0.7, respectively^33^. However, to perform this analysis we needed to determine the physical dimensions of the bacterial flagella, which was done by conventional AFM topographic analysis. Polar bacterial flagella are macromolecular protein complexes which are soft and poorly adhered to the substrate. Therefore, the AFM topographic images, even after tip deconvolution, might not represent the actual geometry of the flagella due to sample deformations by the tip or to small displacements caused during AFM imaging. In order to assess this possibility, we proceed here to reanalyze the experimental results obtained in ref.^33^ from the perspective of the present work. To this end we assume a known dielectric constant for the flagella (~3–5 typical of proteins^31,40^), and then determine the corresponding width and height compatible with the EFM measurements. This example will also serve to illustrate the particularities of the method when applied to low polarization nano-objects.

**Figure 5:**
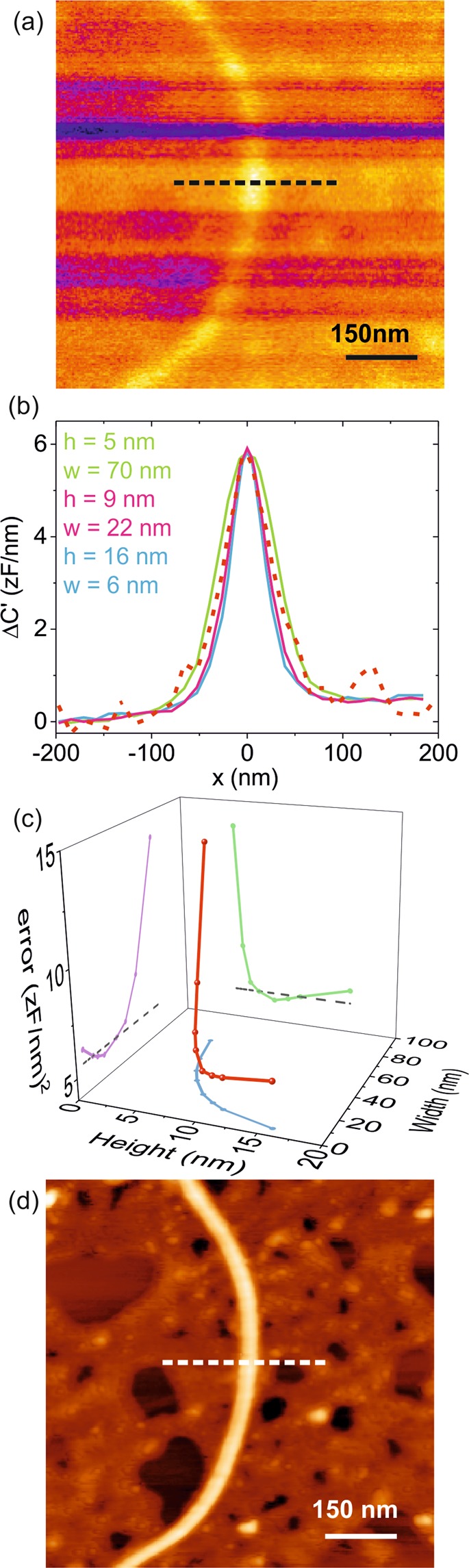
(**a**) EFM image in constant height mode of a bacterial flagellum acquired at a tip substrate distance z = 26.4 ± 0.5 nm. (**b**) (Dashed line) Cross-section profile along the dashed line in (**a**). Note that the contrast scale is in zF/nm. (Continuous lines) Examples of calculated EFM profiles for values of h and w that give the right maximum experimental contrast. Parameters of the calibrated tip geometry used in the calculations: R = 24.5 ± 0.5 nm and θ = 11.5°. (**c**) Cumulative square error R^2^ of the different theoretical profiles with respect to the experimental profile as a function of w and h. The position of the minimum gives the physical dimensions of the flagellum: h_EFM_ = 9 ± 2 nm and w_EFM_ = 22 ± 10 nm. (**d**) AFM topographic image of the flagellum analyzed obtained in gentle dynamic mode. From the image one obtains the physical dimensions h_AFM_ = 9.5 ± 0.5 nm and w_AFM_ = 23 ± 2 nm.

Figure 5a shows a constant height EFM image of one of the bacterial flagella of *S. oneidensis* analyzed in ref.^33^ (flagellum #2). In the EFM image we observe that different lines show slightly different contrast values. These differences do not correspond to a change in the geometry of the flagellum or to a variation of its dielectric constant, but to slightly different imaging tip-substrate distances, due to the presence of some residues on the substrate which introduce slight variations in the reference distance taken at the beginning of each line during the constant height EFM imaging mode^33^.

An EFM approach curve has been acquired on a bare part of the substrate (not shown) to calibrate the tip radius and set the EFM imaging distance, giving *z* = 27 ± 1 nm and *R* = 24 ± 1 nm and *θ* = 11 ± 1°. By assuming a dielectric constant *ε*_*r*_ = 4 for the flagellum and applying the procedure described before, we have determined the possible couples of height/width values compatible with the measured dielectric contrast. For each of the couples we calculated the corresponding EFM profiles (Fig. 5b), from where we calculated the cumulative error *R*^2^ with respect to the experimental profile (red dashed line in Fig. 5b). In the calculus of the profiles we considered the presence of a ~2 nm thin layer of residues on the substrate^33^. A plot of *R*^2^ with respect to the possible widths and heights (Fig. 5c) reveals again the presence of a minimum, which corresponds to the physical dimensions of the flagella that best fit the measured EFM profiles. In the present case we obtained *h*_*EFM*_ = 9 ± 2 nm and *w*_*EFM*_ = 22 ± 10 nm. These physical dimensions are completely realistic for a bacterial flagellum on a substrate^39^ and compare well with the dimensions obtained directly from a gentle topographic AFM image acquired in intermittent contact mode on the same flagellum (Fig. 5d), which gave *h*_*AFM*_ = 9.5 ± 0.5 nm and *w*_*AFM*_ = 23 ± 2 nm. Similar good agreement has been obtained by analyzing additional flagella (see Figs S13 and S14 in Supplementary Information), or additional cross-section profiles (see Fig. S15 in Supplementary Information). We have also shown that the dimensions extracted are robust against small uncertainties on the dielectric constant values (see Fig. S16 in Supplementary Information). These results show that the proposed EFM method can be successfully applied to size small scale fragile low polarizable nanoscale objects, with no need of information coming from its topography.

## Discussion

We have shown that the physical dimensions of single nanoescale objects of known dielectric constant can be determined with good accuracy from the measurement of its electrical polarization by means of EFM measurements combined with a specifically designed quantification multiparameter extraction algorithm. We have shown that the values for the width and height of the nano-objects obtained are comparable to those obtained from AFM topographic imaging, with just a slightly larger uncertainty, especially on what concerns the width. We have shown that the proposed EFM method, being based in a non-contact imaging mode, can be applied to soft and poorly adhered samples, where conventional AFM imaging can face some difficulties.

**Figure 6:**
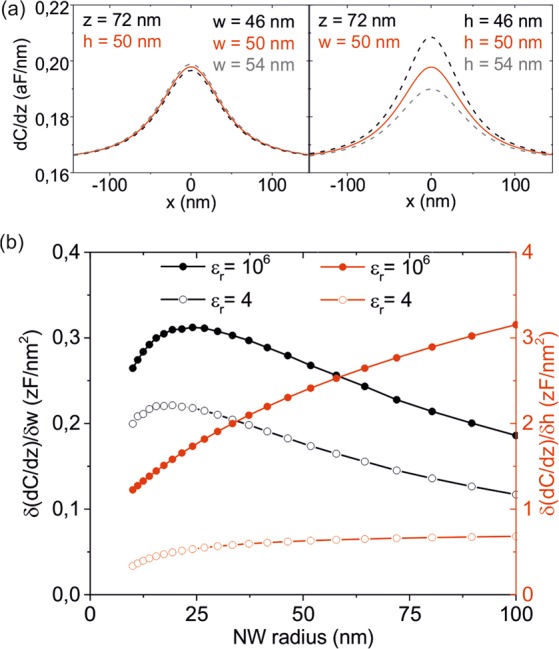
(**a**) (red solid line) Calculated EFM capacitance gradient cross-section profiles for a metallic circular NW of width w = 50 nm at a tip-NW distance z = 22 nm, and for widths (left panel) and heights (right panel) varying ± 4 nm (dashed lines). (**b**) Calculated sensitivity of the EFM capacitance gradient contrast to variations of the width (black symbols, left axis) and of the height (red symbols, right axis) of a circular NW as a function of the NW radius. Solid symbols correspond to ε_r_ ≫ 1 and empty symbols to ε_r_ = 4. Parameters of the simulations, when not otherwise stated: tip radius 31 nm, cone angle 25°, NW length 4000 nm, NW height 50 nm, NW width 50 nm and tip-substrate distance 72 nm.

In the EFM method proposed the height is obtained with better accuracy than the width. This fact is due to the higher sensitivity of the electric polarization force acting on the EFM probe on the height of the nanoscale object than on its width. We illustrate it in Fig. 6a where we show EFM capacitance gradient profiles calculated at a tip-substrate distance *z* = 72 nm for a circular metallic NW of width and height 50 nm (red line), and for the same NW but with widths (left) and heights (right) varied by ± 4 nm (dashed lines).

It is apparent that the variation in the EFM contrast is much larger when the height is varied than when the width is varied. Figure 6b shows this fact in terms of the sensitivity to variations in the width (black symbols, left axis) and height (red symbols, right axis) as a function of the NW radius, for the case of metallic (solid symbols) and low polarizable (*ε*_*r*_ = 4) (empty symbols) circular NWs. We see that the sensitivity to the height is nearly one order of magnitude larger than to the width. Therefore, a higher accuracy can be achieved when determining the height. Furthermore, the sensitivity in both, height and width, is lower for low polarizable NWs, from where the accuracy achievable in these cases would be lower, as we can see by comparing the accuracies for the cases of the metallic AgNWs and for the bacterial flagella. Finally, we note that the sensitivity to the height decreases by reducing the size of the NW, due to a reduction in the signal to noise ratio. Instead, the sensitivity to the width increases as the width increases up to roughly the tip radius, after which it starts decreasing smoothly. These considerations are important in order to select the appropriate tip radius for a given characteristic size of the nano-object to be analyzed.

On the other hand, the accuracy with which the physical dimensions can be determined depends also on the noise of the EFM measurements, as we mentioned before (dashed lines in Fig. 3g). In the present work, the experimental noise of a single EFM profile measured on the AgNWs was ~1−2 zF/nm, which we reduced to ~ 0.5 zF/nm by averaging five consecutive profiles. Experimentally it has been shown that a noise level down to just ~ 0.1 zF/nm can be achieved^30^. Since the uncertainty scales roughly proportionally to the experimental noise we expect that the uncertainty of the extracted physical dimensions can still be further reduced. This reduction will, at some point, be overcome by the appearance of other sources of uncertainty, such as those related to the tip geometry calibration or the determination of the tip-sample distance. In any case, it is expected that the accuracy achievable with the EFM method under optimal conditions will be close to the one achievable by conventional AFM topographic imaging.

One obvious drawback of the proposed EFM method is the need to know the dielectric constant of the nano-object *a priori*. We have investigated the possibility to overcome this limitation by considering a multiparametric extraction algorithm that included the dielectric constant of the nano-object as an additional unknown parameter to be determined, together with the nano-object dimensions. We concluded that, from the mathematical point of view, such an algorithm can be solved and provides a unique solution. However, for its practical implementation it needs a noise floor well below ~ 0.1 zF/nm, which is very difficult to attain in practice (see ref.^37^ for a similar discussion in the context of EFM nanotomographic reconstruction). Therefore, even if theoretically possible, the implementation of this more general approach in practice will need to await until significant instrumental noise improvements can be attained.

Finally, we mention that the method developed is sensitive to the cross-section shape of the nanowire object only in an effective manner. Indeed, we have shown that due to tip convolution effects, the shape of the cross-section of the object (e.g. circular, square, pentagon, hexagon, etc.) can not be directly inferred (see Fig. S17), since all of them show a rounded shape. But, in all cases, the cross-section profiles can be interpreted in terms of a nanowire with an elliptical shape, where the height is close (within ~1 nm) to the actual height of the nanoscale object, and the width represents an effective value, the larger the further the shape departs from an actual ellipsoidal shape (see Table S2 in the Supplementary Information).

## Materials and Methods

### Silver nanowires sample preparation

We used commercial ~50 nm diameter silver nanowires A50, research grade from Novarials. The sample contains 0.5 g of AgNWs suspended in 50 ml of isopropanol (IPA) for a final concentration of 10 mg/ml. We diluted the AgNWs in IPA 20 times and we put one drop over the substrate used for the measurements. The substrates used are TEM and AFM/EFM/SEM finder grids (see below) and Highly Oriented Pyrolytic Graphite (HOPG).

### AFM/EFM/SEM custom finder grid

To analyze the same nanowire in AFM, EFM and SEM imaging we fabricated a custom-made finder grid in the Nanotechnology Platform of IBEC, Spain. The grid has been fabricated on a highly doped silicon wafer (76.2 mm diameter, Boron p-doped, orientation <100> and resistivity 0.001–0.005 Ω/cm, from UniversityWafer, Inc). It consists of ~ 200 × 200 labelled 50 × 50 μm^2^ square areas delimited by a gold square stripe 5 μm wide and 50 nm thick, plus 10 nm of Cr adhesion layer. Each square is labelled with gold microfabricated letters and numbers (from AA1 to ZZ200) of an approximate size 50 × 50 μm^2^ easily identifiable with the integrated optical camera of the AFM or with the SEM. The grid, being fabricated on a highly doped (conductive) support, facilitates both AFM and EFM imaging, as well as, SEM imaging (see Fig. S7 in Supplementary Information).

### Transmission electron Microscopy imaging and width determination

TEM images have been obtained with a Jeol J1010 80 kV TEM, from the Scientific Services of the University of Barcelona (Spain). We used a 200 mesh and 3.06 mm diameter commercial copper TEM finder grid (Monocomp), covered with a formvar layer and 7 nm of carbon (see Fig. S1 in Supplementary Information). To determine the width of the nanowire we acquired images of 1.8 × 1.3 μm^2^ for the ensemble analysis of the sample and of 1.3 × 1.0 μm^2^ for the individual measurements, both with 1376 × 1032 pixels. The width of the nanowires was obtained by using the software Fiji by Image J. For each nanowire five different determinations have been done, from where the mean and the standard deviation of the measurements were obtained.

### Scanning electron microscopy imaging and width determination

We used a Nova NanoSEM FEI SEM using the detector LVD from the Nanotechnology platform of the Institute of Bioengineering of Catalonia (IBEC), Spain. Images have been obtained by applying a voltage of 5 kV, a current of 56 pA and a working distance of 6.5 mm. SEM images have been performed on AgNW samples prepared on both the TEM and the AFM/EFM/SEM custom made finder grids. To determine the width of a nanowire we acquired images of 2.5 × 2.2 μm^2^ (2048 × 1886 pixels). The widths of the AgNWs have been determined as described above for the TEM images.

### Atomic Force microscopy imaging and height determination

We used a Cypher S AFM from Oxford Technologies (former Asylum Research). Topographic AFM images of 1024 × 1024 pixels (6.4 × 6.4 μm^2^) have been obtained at 0.5 Hz per line in intermittent contact mode by using conductive PtSi-CONT probes (Nanosensors) with a spring constant *k* ∼ 0.2 N/m (determined by the provider according to the probe dimensions), resonance frequency *f*_*r*_ ∼ 13 kHz, nominal tip radius *R* ∼ 20 nm and half cone angle *θ* ∼ 11.5°. AFM images have been obtained on samples prepared on the different substrates (TEM and AFM/EFM/SEM finder grids and HOPG). To determine the height of a nanowire we acquired images of 0.5 × 0.5 μm^2^ (256 × 256 pixels), and for each nanowire five different determinations have been done from consecutive topographic profiles, from where the mean and the standard deviation of the measurements were obtained. Images were processed in both Gwyddion and WSxM^41^ (Nanotec Electrónica S.L.).

### Tip geometry calibration

The tip geometry was determined from EFM approach curves measured on a bare part of the metallic substrate, as detailed elsewhere^30,38^. We have verified that determining the tip geometry from EFM measurements offers similar results than alternative methods based on SEM imaging, the use of calibration samples or the use of tip reconstruction methods^19–24^ (see Figs S3 and S4 in Supplementary Information). The following tip parameters were set to their nominal values in the tip geometry calibration: cone height *H* = 12.5 μm, local cantilever *L*_*c*_ = 3 μm and cantilever thickness *W*_*c*_ = 3 μm.

### Tip deconvolution and width determination from AFM imaging

The deconvoluted width of the AgNWs and of the bacterial flagella has been obtained from the topographic AFM images by means of a semi-automatic analytical tip deconvolution model. From the AFM topographic images, we obtained a cross-section profile by using the Gwyddion free software. Then, through a Matlab script the profile in the *x* axis was centered and moved to *h* = 0 nm from the substrate region. Then, the Full Width at Half Maximum (FWHM) and the height (maximum point of the profile) were determined. To calculate the FWHM of the profile, the script adjusts a polynomic function of second order. To obtain the deconvoluted width, the scripts solves the following system of equations, which describes the convoluted profile of a conical tip ended by a tangent sphere and an ellipse, (see Fig. S5 in Supplementary Information)

1 $$\begin{array}{c}{p}_{y}\le {y}_{0}\\ \{\begin{array}{l}\frac{{(w-{x}_{0})}^{2}}{{a}^{2}}+\frac{{y}_{0}^{2}}{{b}^{2}}=1\\ {y}_{0}={p}_{y}+({x}_{0}-{p}_{x})\tan (\frac{\pi }{2}-\theta )\\ \tan (\frac{\pi }{2}-\theta )=\frac{{b}^{2}}{{a}^{2}}\frac{(w-{x}_{0})}{{y}_{0}}\end{array}\end{array}$$

2 $$\begin{array}{c}{p}_{y} > {y}_{0}\\ \{\begin{array}{l}\frac{{(w-{x}_{0})}^{2}}{{a}^{2}}+\frac{{y}_{0}^{2}}{{b}^{2}}=1\\ {x}_{0}^{2}+{({y}_{0}-R)}^{2}={R}^{2}\\ \frac{{x}_{0}}{({y}_{0}-R)}=\frac{{b}^{2}}{{a}^{2}}\frac{(w-{x}_{0})}{{y}_{0}}\end{array}\end{array}$$

where *x*_0_, *y*_0_ is the point at which the tip contacts the ellipse when the tip is at the half-height point; *a*, *b* are the semiaxes of the ellipse; *w* = FWHM/2 is the distance between the center of the ellipse and the center of the tip; *R* and *θ* are the radius and the half cone angle of the tip, respectively and *p*_*x*_ = *R cos(θ)* and *p*_*y*_ = *R − R sin(θ*). The radius *R* and the angle *θ* of the tip are known from the tip calibration, while the height is 2*a* and it is known from the topographic profile, so that the remaining unknowns are *b, x*_0_ and *y*_0_. This procedure is repeated for five nearby profiles and the mean and standard deviation corresponds to the AFM width and error, respectively.

### Electrostatic force microscopy imaging

EFM images have been obtained with the AFM system and probes described above. We recorded the 2ω harmonic of the probe oscillation amplitude, *A*_2*ω*_, by using the system internal lock-ins in the two-pass SNAP mode, the Cypher built-in line by line constant height mode, as detailed in previous works^30^. EFM data were obtained with a voltage amplitude *v*_*ac*_ = 5 V at a frequency *f* = 2 kHz (well below the mechanical resonance peak of the probe). EFM measurements have been performed on the AFM/EFM/SEM finder grid. EFM data were reported in terms of the capacitance gradient, which is related to the 2ω oscillation amplitude by^30^

3 $$\frac{dC}{dz}=\frac{2\sqrt{2}}{{{v}_{rms}}^{2}}k\frac{({A}_{2\omega }-{A}_{2\omega ,offset})}{mG}$$

where *k* is the equivalent spring constant of the cantilever, *v*_*rms*_ the rms voltage amplitude, *A*_2*ω,offset*_, is the lock-in offset, *m* the optical lever sensitivity and *G* the lock-in gain. Typical values of these parameters are *m* ~ 1 mV/nm, *A*_2*ω,offset*_ ~ 0.1 mV, *G* = 1, *v*_*rms*_ ~ 3 V, *k* ~ 0.15 N/m and *A*_2*ω*_ ~ 10–20 mV. The capacitance gradient instrumental noise was in the range 1–2 zF/nm depending on the probe used and recording parameters. EFM measurements were performed in controlled dry air conditions (RH < 1%) maintained by a N_2_ flow.

### Finite element numerical calculations

We used finite element numerical calculations to simulate theoretical EFM capacitance gradient profiles and approach curves for the tip nanowire system, as in earlier works^30,36^. The geometrical model and theoretical approach are the same as those detailed in ref.^33^, adapted to the dimensions of the AgNWs and tips used in the present work.

### Flagella sample preparation and AFM and EFM flagella imaging

The experimental EFM and AFM data for the flagella have been obtained from ref.^33^, to which we refer for details. In the present work we performed a reanalysis of those data corresponding to flagella #1, 2 and 5 from *S. Oneidensis* and to the flagellum from *P. Aeruginosa*, but from the perspective of the present work, i.e., from the perspective of extracting the physical dimensions from electric polarization EFM measurements. Also, different sections of the flagellum have been analyzed in this work, giving rise to slightly different values (variations of 1–2 nm) within the uncertainty of the measurements.

## Conclusions

In this work, we have proposed a method to size single nanoscale objects based on the measurement of its electrical polarization by using Electrostatic Force Microscopy measurements combined with a multiparameter quantification extraction algorithm. We have validated the method for the case of ~50 nm diameter silver nanowires and obtained values for the width and height comparable to those obtained from (deconvoluted) AFM topographic imaging and TEM imaging, with just a slightly smaller accuracy. As an application of the method to a fragile and low polarizable nano-object we have considered the case of a macromolecular protein complex (bacterial polar flagella) ~10 nm in diameter, providing again a good agreement with AFM topographic data. The main advantage of the proposed method is that it can be applied to soft and poorly adhered samples, as it is based on the measurement of the long-range electric polarization forces, which does not require the contact with the sample at any moment. This fact opens interesting possibilities in the determination of the physical dimensions of nano-objects made of soft materials like biological materials, polymers, gels, biomolecules, liquids, etc., for which an accurate determination has remained elusive for Scanning Probe Microscopy methods until now.

## Supplementary information

Supplementary Material

## Data Availability

All data generated or analyzed during this study are included in this manuscript and its supplementary information files, or they are available upon request.
