# Supplementary material for: Sizing single nanoscale objects from polarization forces
Source: Sci Rep. 2019 Oct 2;9:14142. doi: 10.1038/s41598-019-50745-5 (PMC6775056; doi:10.1038/s41598-019-50745-5)
Supplement: Supplementary file 1 — Supplementary Material [file 41598_2019_50745_MOESM1_ESM.pdf]

Supplementary Information

for

Sizing single nanoscale objects from polarization forces

H. Lozano[1], R. Millan-Solsona[1,2], R. Fábregas[1,2], G. Gomila[1,2]*

[1] Nanoscale Bioelectrical Characterization, Institute for Bioengineering of Catalonia (IBEC), The Barcelona Institute of Science and Technology (BIST), c/ Baldiri i Reixac 11-15, 08028, Barcelona, Spain

[2] Departament d'Enginyeria Electrònica i Biomèdica, Universitat de Barcelona, C/ Martí i Franqués 1, 08028, Barcelona, Spain

**Corresponding author: ggomila@ibecbarcelona.eu*

S1. TEM finder grid

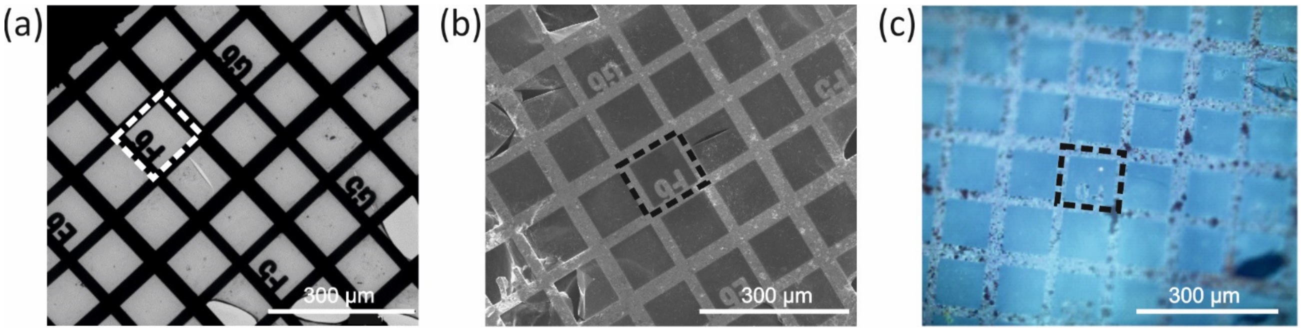

Figure S1: (a), (b) and (c) TEM, SEM and Optical Microscopy images of the finder TEM grid, with one of the cells used in the experiments of the main text highlighted by a dashed line. The optical microscopy image has been obtained with the camera integrated in the AFM and which is used to position the tip in the selected area.

S2. Width determination from TEM and SEM images

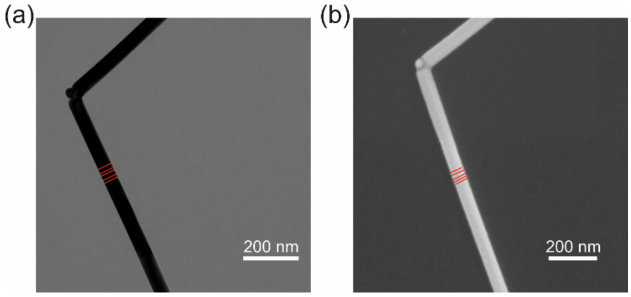

Figure S2: (a) TEM and (b) SEM zoom-in images of nanowire #1 in Figures 1b and 1c of the main paper. The segments indicate the five widths measured on the nanowire to obtain the average width. In this case we obtained $w_{SEM} = 63 \pm 2$ nm and $w_{TEM} = 52.5 \pm 0.7$ nm.

S3. EFM and SEM tip geometry calibration

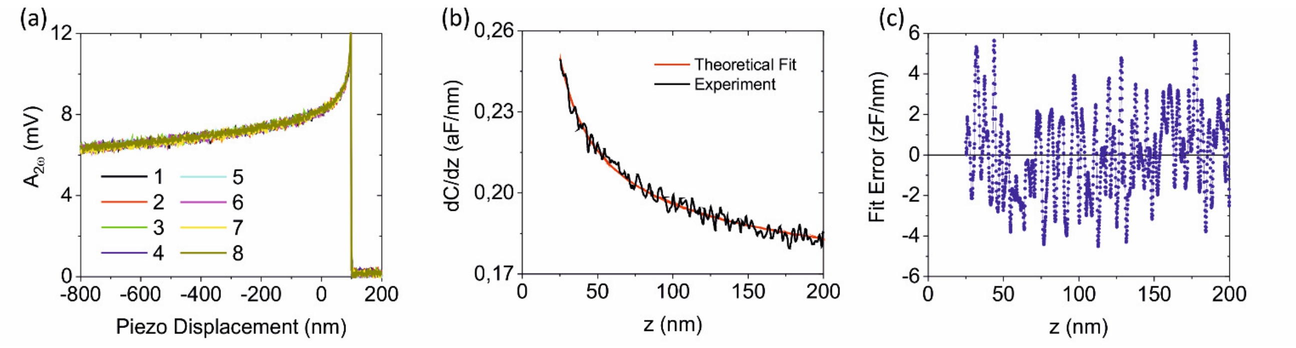

Figure S3: (a) Raw 2ω oscillation amplitude EFM approach curves measured before and after an experiment on a bare metallic part of the sample, showing the stability of the measuring tip. (b) Theoretical fitting (red line) to one of the electrical approach curves already calibrated (in capacitance gradient units) (black line). The extracted parameters are $R = 33 \pm 1$ nm, $\theta = 11 \pm 1°$ and $C'_{offset} = 117 \pm 2$ zF/nm. The remaining parameters of the tip have been set to their nominal values $H = 12.5$ µm and $W_c = L_c = 3$ µm. (c) Error of the electrical approach curve fitting, giving a mean = 0.3 zF/nm and a standard deviation = 2.1 zF/nm.

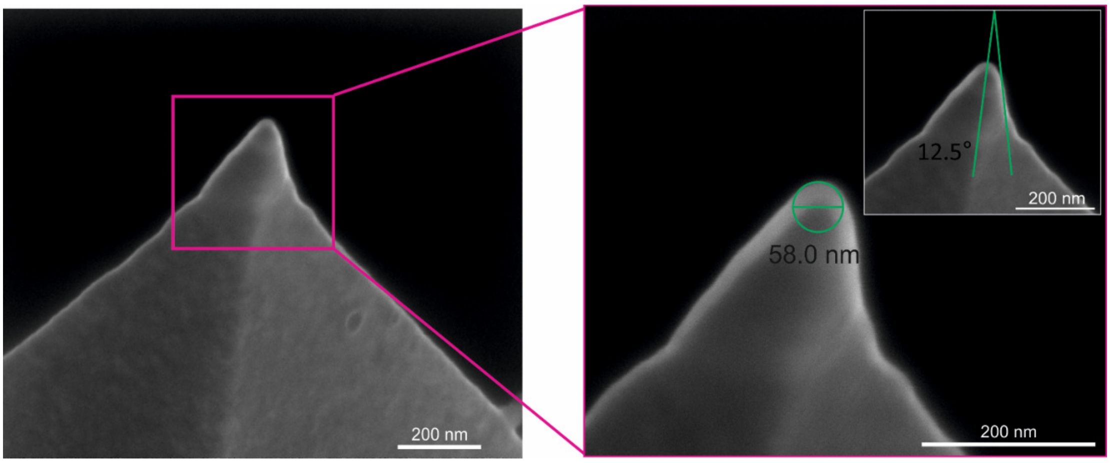

Figure S4: SEM images of one of the tips used in the study. From the images we obtain R = 29 nm and θ = 12.5°, in good agreement with the EFM calibration of the same tip shown in Figure S3.

S4. AFM tip deconvolution

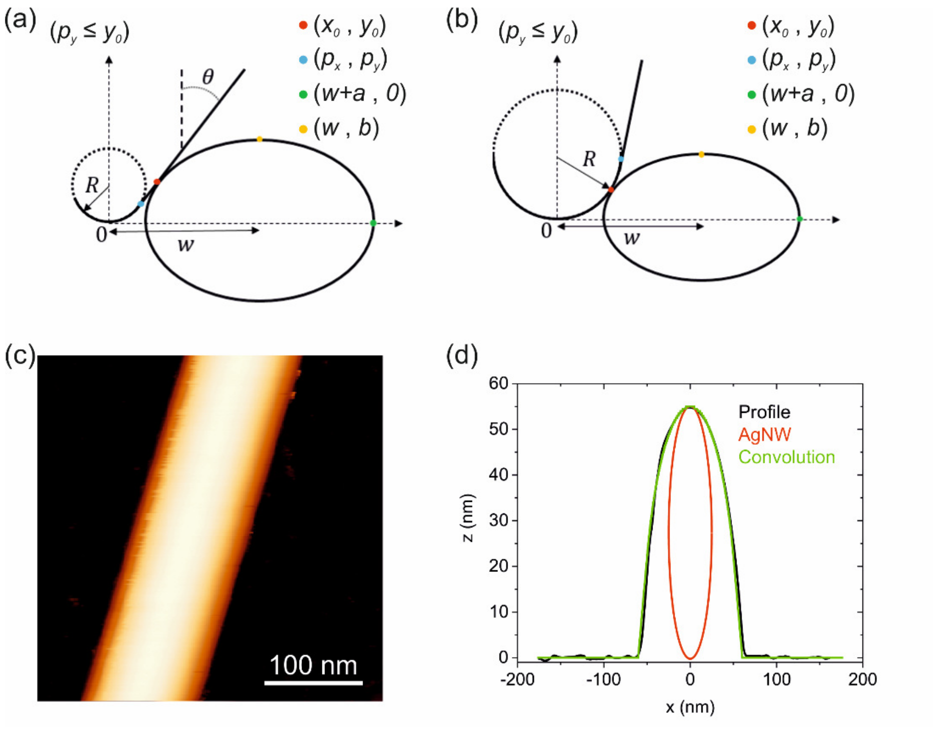

Figure S5: (a) and (b) Schematic representations of the geometrical model used to calculate the tip convoluted profile for the case of a nanowire with elliptical cross-section and a tip consisting of a cone with a tangent sphere. (c) AFM image of AgNW #1 in Figure 1. (d) Tip deconvolution analysis, providing a width $w_{AFM} = 50 \pm 2$ nm.

S5. Population analysis of the AgNW sample by SEM, TEM and AFM

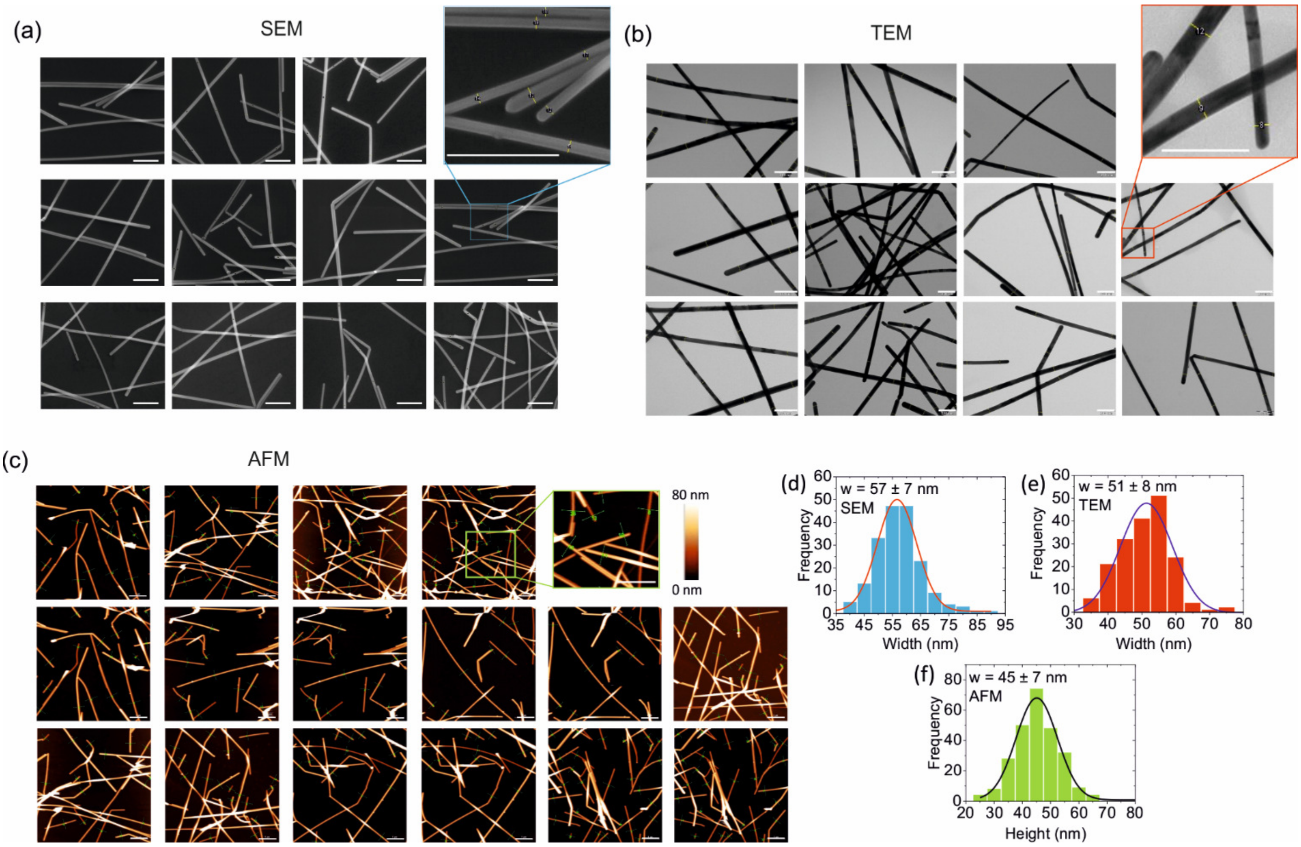

Figure S6: (a) SEM, (b) TEM and (c) AFM images of different populations of AgNWs. The images correspond to different regions and even samples (TEM images are taken on TEM grids, while the SEM and AFM images on HOPG). The scale bars are: 500 nm in (a), 200 nm in (b) and 1 µm in (c). (d) and (e) represent the histogram of the statistical distribution of the widths measured from the SEM and TEM images, and (f) of the heights measured in the AFM images. The mean values and standard deviations are indicated in each figure.

Technique	*2xN*	Mean (nm)	Standard deviation (nm)	Maximum (nm)	Minimum (nm)
TEM	182	48	7	72	31
SEM	182	55	8	86	36
AFM height	268	46	8	73	25
AFM width	268	50	11	81	19

Table S1: Summary of the data obtained from the images in Figure S4.

S6. Custom made AFM/EFM/SEM finder grid

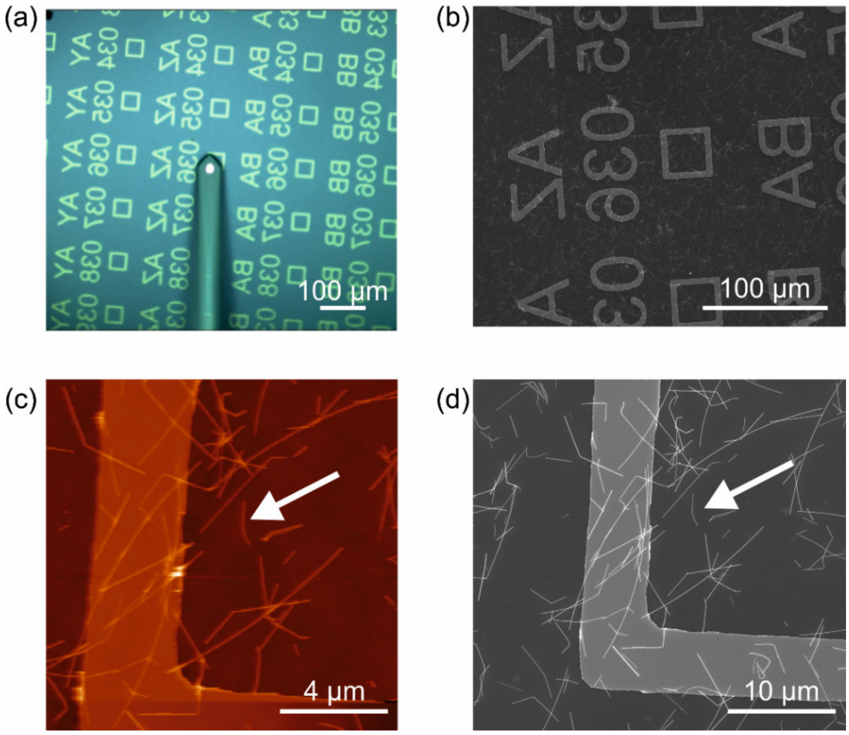

Figure S7: Optical and (b) scanning electron microscopy images of the microfabricated AFM/EFM/SEM custom finder grid. In (a) the AFM probe is also shown. (c) AFM and (d) SEM image of the same area of the custom-made AFM/EFM/SEM finder grid with AgNWs on it, enabling to analyze the same AgNW (highlighted with an arrow) by AFM, EFM and SEM.

S7. Details on the theoretical calculations of the dielectric profiles

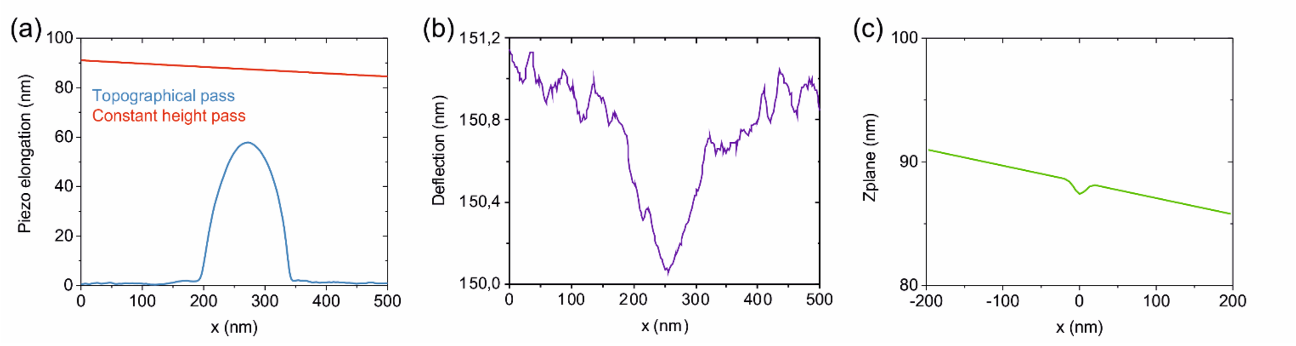

Figure S8: (a) Piezo elongation in the AFM topographic pass (blue line) and in the EFM constant height pass (red line). (b) Cantilever deflection profile acquired simultaneously to a constant height EFM profile. The combination of the two profiles obtained during the constant height scan gives the actual position of the tip apex end during the constant height scan, which is the one used in the calculations of the theoretical profiles.

S8. SEM image of the AgNW analyzed in Fig. 3 of the main paper.

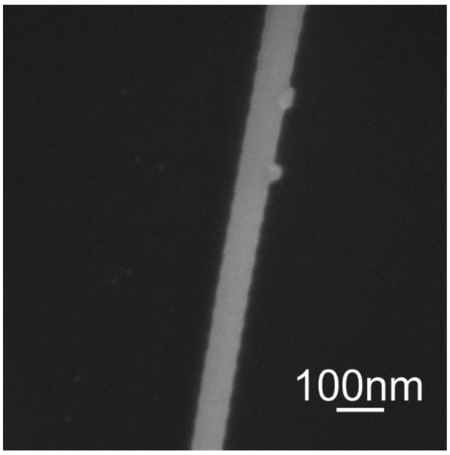

Figure S9: SEM image of the AgNW analyzed in Figure 3, giving a width of w_{SEM} = 67 ± 2 nm.

S9. Data on the additional AgNWs reported in Fig. 4.

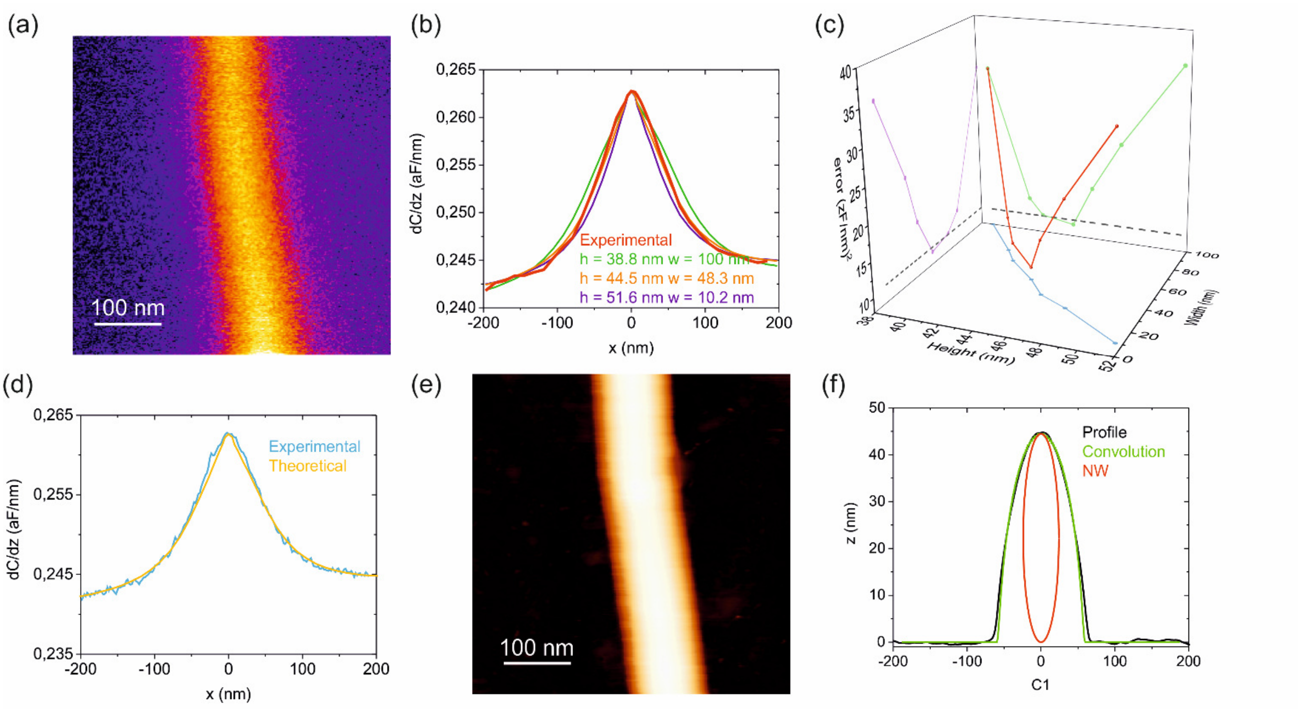

Figure S10: (a) Constant height EFM image of AgNW #2 corresponding to Fig. 4 of the main paper. The imaging distance has been set to $z = 77.5$ nm by using an EFM capacitance gradient approach curve (not shown). With the same curve the tip geometry has been determined giving $R = 35 \pm 1$ nm, $\theta = 24 \pm 1°$, $C'_{offset} = 105 \pm 2$ zF/nm. (b) Calculated EFM cross-section profiles for some width/height couples that give the experimentally measured contrast. (c) Cumulative error square of the calculated profiles with respect to the experimental profile as a function of the width and height. From the position of the minimum we obtain $w_{EFM} = 55 \pm 10$ nm and $h_{EFM} = 43 \pm 1$ nm. (d) Comparison of the selected EFM theoretical profile with the experimental one. (e) AFM image of the same AgNW. (f) Fitted convoluted AFM topographic profile to the experimental one giving $w_{AFM} = 48 \pm 1$ nm and height $h_{AFM} = 44 \pm 1$ nm. From a SEM image (not shown) we obtained $w_{SEM} = 55 \pm 2$ nm.

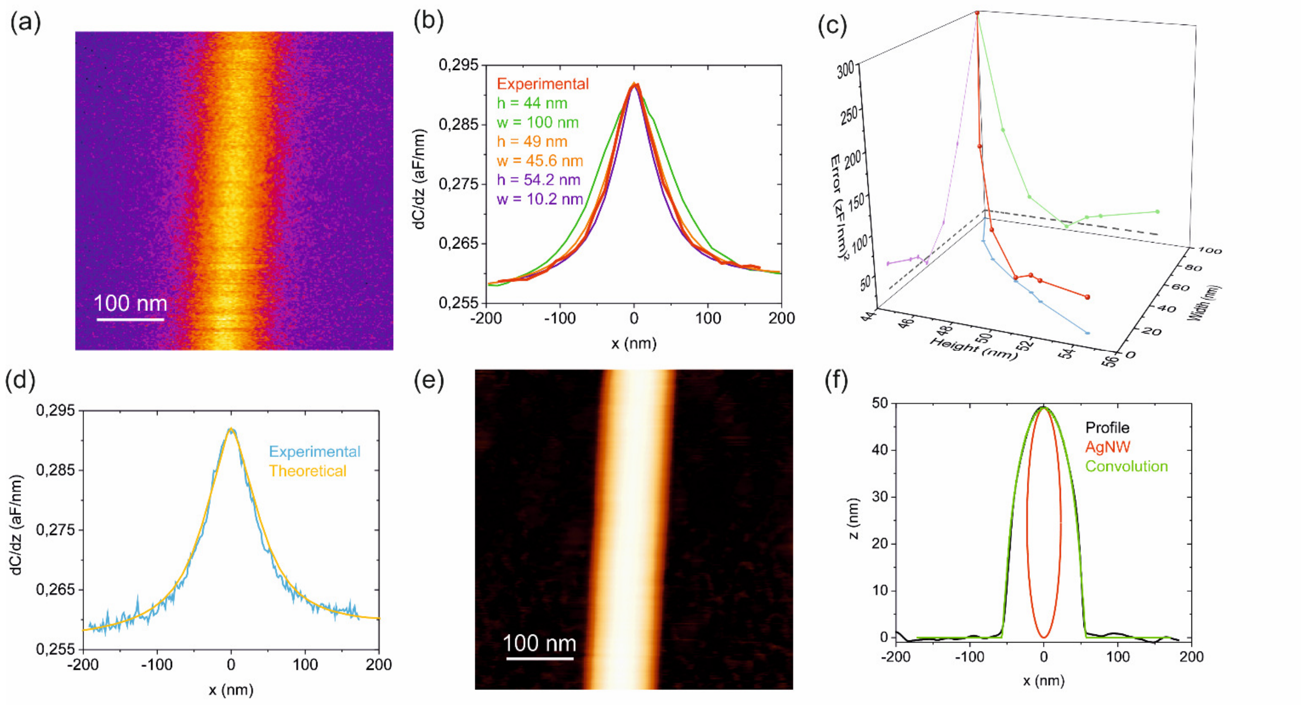

Figure S11: (a) Constant height EFM image of AgNW #3 corresponding to Fig. 4 of the main paper. The imaging distance has been set to z = 70 nm by using an EFM capacitance gradient approach curve (not shown). With the same approach curve the tip geometry has been determined giving $R = 31 \pm 2$ nm, $\theta = 25 \pm 1°$ and $C'_{offset} = - 112 \pm$ zF/nm. (b) Calculated EFM cross-section profiles for some couples of width/height that give the experimentally measured contrast. (c) Cumulative error square of the calculated profiles with respect to the experimental profile as a function of the width and height. From the position of the minimum we obtain $w_{EFM} = 42 \pm 3$ nm and $h_{EFM} = 49 \pm 1$ nm. (d) Comparison of the selected EFM theoretical profile with the experimental one. (e) AFM image of the same AgNW. (f) Fitted convoluted AFM topographic profile to the experimental one giving $w_{AFM} = 46 \pm 2$ nm and height $h_{AFM} = 49.0 \pm 0.5$ nm. From a SEM image (not shown) we obtained $w_{SEM} = 65 \pm 2$ nm.

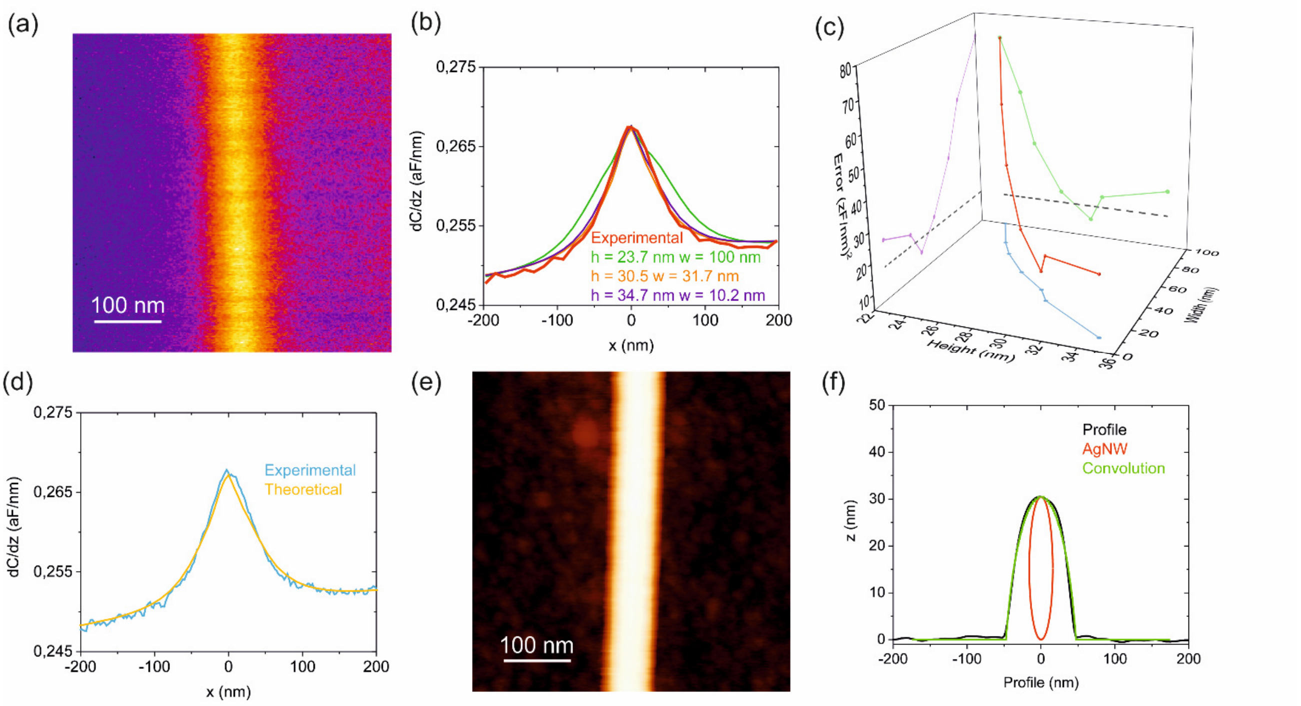

Figure S12: (a) Constant height EFM image of AgNW #4 corresponding to Fig. 4 of the main paper. The imaging distance has been set to z = 60 nm by using an EFM capacitance gradient approach curve (not shown). With the same curve the tip geometry has been determined giving R = 36 ± 1 nm, θ= 25 ± 1°, C'_{offset} = 99 ± 2 zF/nm. (b) Calculated EFM cross-section profiles for couples of width/height that give the experimentally measured contrast. (c) Cumulative error square of the calculated EFM profiles with respect to the experimental profile as a function of the width and height. From the position of the minimum we obtain w_{EFM} = 41 ± 8 nm and h_{EFM} = 29 ± 1 nm. (d) Comparison of the selected EFM theoretical profile with the experimental one. (e) AFM image of the same AgNW. (f) Fitted convoluted AFM topographic profile to the experimental one giving w_{AFM} = 32 ± 2 nm and height h_{AFM} = 30.5 ± 0.5 nm.

S10. Additional flagella analyzed with the EFM method.

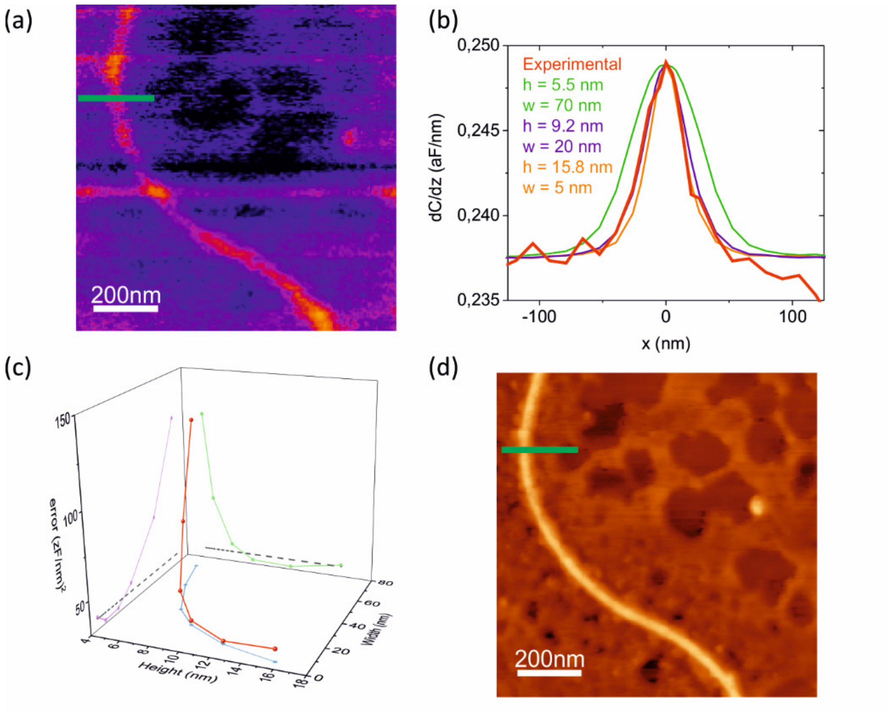

Figure S13: (a) Constant height EFM image of a S. oneidensis flagellum corresponding to flagellum #1 from Ref. (33). The imaging distance was set to z = 21 nm by using an EFM capacitance gradient approach curve (not shown). With the same curve the tip geometry was determined, giving R = 24 ± 1 nm, θ= 11±1° and C'_{offset} = 118 ± 1 zF/nm. (b) Calculated EFM cross-section profiles for couples of width/height that give the experimentally measured contrast. (c) Cumulative error square of the calculated EFM profiles with respect to the experimental profile as a function of the width and height. From the position of the minimum we obtain w_{EFM} = 15 ± 9 nm and h_{EFM} = 12 ± 3 nm. (d) AFM image of the same flagellum. From the analysis of the image we obtain w_{AFM} = 18 ± 2 nm and h_{AFM} = 11 ± 0.5 nm.

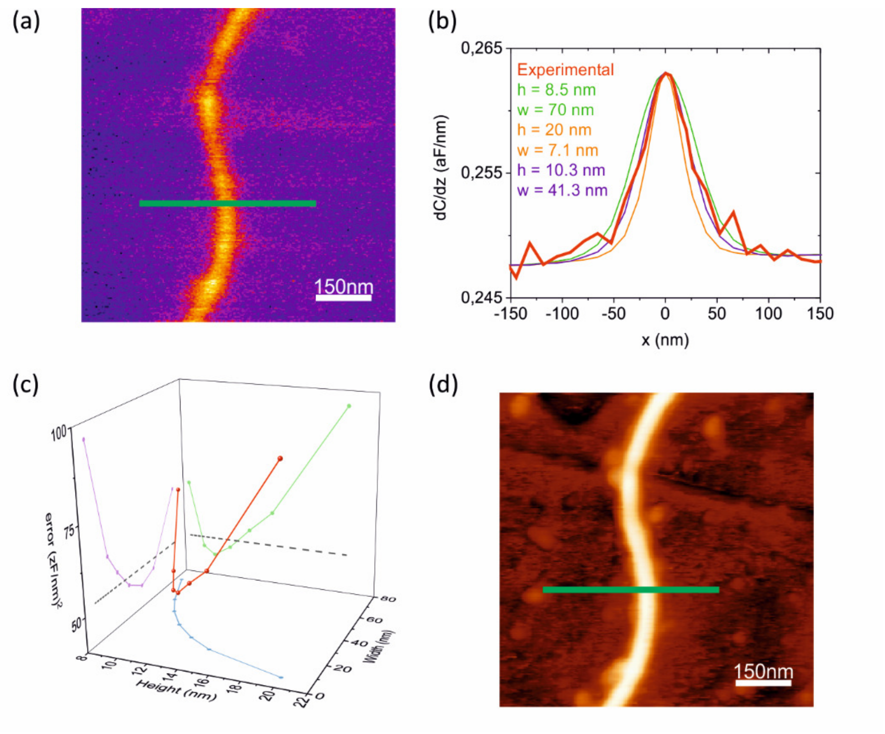

Figure S14: (a) Constant height EFM image of the P. Aeruginosa flagellum reported in Ref. (33). The imaging distance was set to z = 25 nm by using an EFM capacitance gradient approach curve (not shown). With the same curve the tip geometry was determined, giving R = 30 ± 1 nm, θ= 11±1° and C'_{offset} = 128 ± 2 zF/nm. (b) Calculated EFM cross-section profiles for couples of width/height that give the experimentally measured contrast. (c) Cumulative error square of the calculated EFM profiles with respect to the experimental profile as a function of the width and height. From the position of the minimum we obtain w_{EFM} = 41 ±13 nm and h_{EFM} = 11 ±2 nm. (d) AFM image of the same flagellum. From the analysis of the image we obtain w_{AFM} = 35 ± 2 nm and h_{AFM} = 13 ± 1 nm.

S11. Additional data for Fig. 5

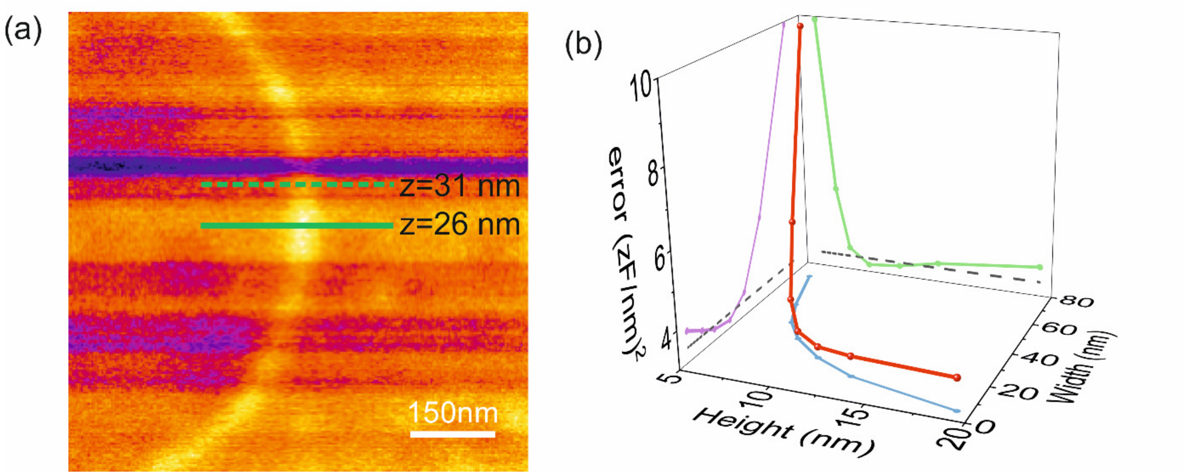

Figure S15. Figure 5a of the manuscript showing an additional cross-section line (dashed line) corresponding to a tip-sample distance of 31 nm. (b) Cumulative square error R^2 of the different theoretical profiles with respect to the experimental profile as a function of w and h. The position of the minimum gives the physical dimensions of the flagellum: $h_{EFM} = 10 \pm 2$ nm and $w_{EFM} = 24 \pm 8$ nm. These values agrees with the ones reported in the manuscript and obtained from the capacitance gradient profile corresponding to the solid line at a tip sample distance of 26 nm ($h_{EFM} = 9 \pm 2$ nm and $w_{EFM} = 22 \pm 10$ nm).

S12. Effect of the dielectric constant value on the extracted dimensions of the bacterial flagella.

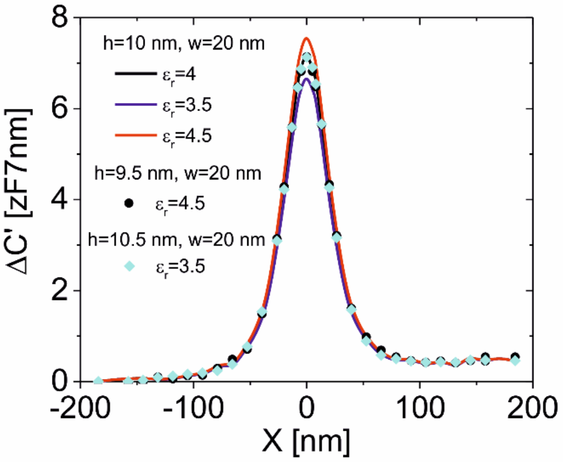

Figure S16: (continuous lines). Numerically calculated capacitance gradient profile for a flagellum with dimensions h=10 nm and w=20 nm and dielectric constants ε_r=3.5, 4 and 4.5. The change in dielectric constant modifies essentially the maximum value of the peak but not its width. (symbols) Capacitance gradient profiles corresponding to flagella with heights h=9.5 nm and 10.5 nm (and width w=20 nm) and dielectric constants ε_r=3.5 and 4.5, matching the profile for h=10 nm, w=20 nm and ε_r=4.

S13. Effect of the cross-sectional geometry of the nanowire on the extracted dimensions.

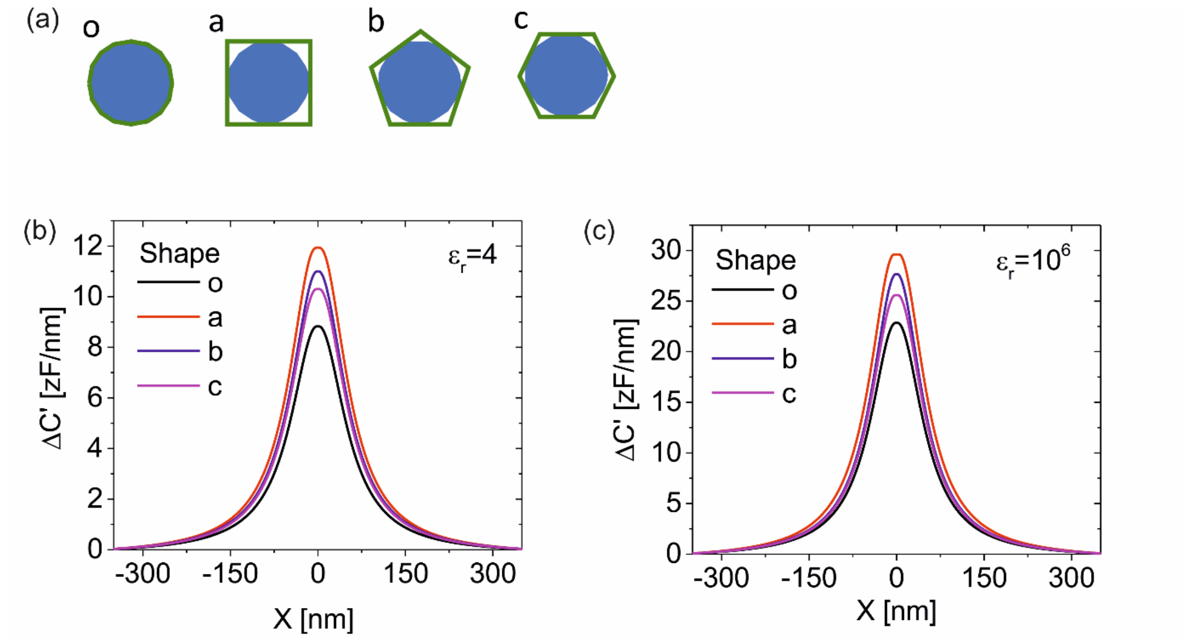

Figure S17. (a) Cross-section shapes of different nanowires (circular, square, pentagon, hexagon), with the circular nanowire inscribed inside. (b) and (b) Capacitance gradient cross-section profile contrasts corresponding to the nanowires with the different cross-section shapes shown in (a) for two different dielectric constants ε_r=4 and ε_r=10^6, respectively. The different cross-section shapes give rise to slightly different capacitance gradient contrast values due, mainly, to the variation in the exposed area in the top part. Parameters: R=35 nm, θ=20º, W=L=3000 nm, H=12500 nm, h=w=50 nm, z=30 nm.

Cross-section shape	H [nm] ε_r=4	W [nm] ε_r=4	H [nm] ε_r=10^6	W [nm] ε_r=10^6
Circular (o)	50	50	50	50
Square (a)	50	70	50	84
Pentagon (b)	51	61	50	70
Hexagon (c)	51	60	50	67

Table S2. Equivalent dimensions for nanowires with different cross-section shapes when analyzed with an elliptical shape model.
